# Nanofertilizers in Modern Agriculture: A Technological Revolution in Plant Nutrition and Resource Efficiency

**DOI:** 10.1021/acsomega.5c08087

**Published:** 2025-11-21

**Authors:** Moacir C. do Couto Junior, Leandro I. da Silva, Mariana de S. Ribeiro, Caroline Dambroz, Tatiana C. e Bufalo, Marcelo Pedrosa Gomes, Joyce Dória

**Affiliations:** † Department of Agriculture, 67739 Federal University of Lavras (UFLA), Lavras 37203-202, Brazil; ‡ Department of Biology, Federal University of Lavras (UFLA), Lavras 37203-202, Brazil; § Department of Physics, Federal University of Lavras (UFLA), Lavras 37203-202, Brazil; ∥ Department of Botany, Biological Sciences Sector, Federal University of Paraná, Avenida Coronel Francisco H. dos Santos, 100, Polytechnic Center Jardim das Américas, 81531-980 Curitiba, Paraná, Brazil

## Abstract

Agriculture as we know it today has been made possible by the modernization of management practices and technological advances in agricultural inputs. However, the excessive, continuous, and inappropriate use of these inputs has led to nutrient loss, negatively affecting the ecosystem and human health. Population growth and climate change have driven the search for alternative technologies that increase crop productivity and reduce environmental impacts. Nanotechnology has emerged as a promising tool in agriculture, promoting better nutrient supply, controlled fertilizer release, and greater application and absorption efficiency. Nanofertilizers contribute to increasing crop yields, reducing nutrient losses, and promoting sustainability. This review aims to explore the current literature on nanofertilizers, their properties, synthesis methods, absorption and transport mechanisms, and the interaction between soil and microbiota. It provides an assessment of their agronomic benefits and potential environmental risks. In addition, we present a bibliometric analysis that identifies global trends in the scientific literature focused on nanofertilizers. This study highlights recent advancements and the existing perspective on nanofertilizers while emphasizing the need for regulatory frameworks and long-term field evaluations to ensure their safe and effective implementation in agriculture.

## Graphical abstract

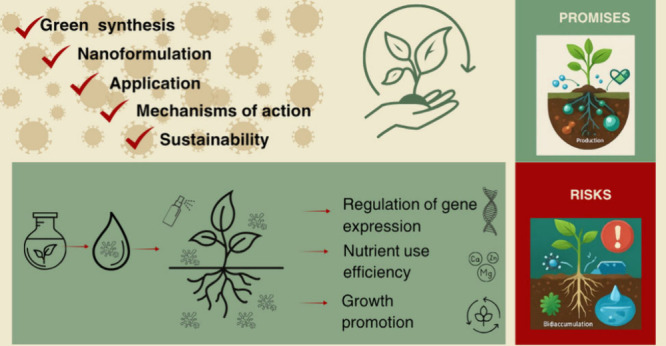

## 1 Introduction

With global population growth, the pursuit of efficient and sustainable agriculture has become a pressing reality worldwide. Furthermore, climate change has driven the search for technologies and management alternatives that enhance crop productivity while reducing environmental impact. The adverse conditions to which plants have increasingly been exposed, such as drought, heat, high light intensity, and salinity resulting from climate change, can interact and negatively affect plant performance, even if the impact of each condition individually is negligible.^1^ In addition, Ishfaq et al.^2^ stated that under environmental stress conditions, such as heat and drought, the uptake and subsequent utilization of nutrients from the soil via the roots may be impaired. These unusual circumstances demand efficient, whether established or innovative, strategies for supplying nutrients to plants.

Modern agriculture, as it is known today, has become possible only through the advancement of management practices and technological evolution of agricultural inputs. Chemical pesticides, fertilizers, inoculants, high-yield genetically modified varieties, machinery, and implements have served as the foundation for the development of monoculture-based agricultural systems.^3^ However, the excessive, continuous, and often improper use of these inputs has negatively impacted ecosystems and consumer health.^4^ Conventional fertilizers are particularly inefficient; up to 50–70% of the applied nitrogen can be lost through volatilization, leaching, and runoff,^5^ contributing to eutrophication and soil degradation. With the growing reliance on synthetic chemical inputs, agricultural systems have become increasingly specialized and simplified, leading to a reduction in the complexity of natural ecological interactions.^3^

In the search for sustainable and economically viable agricultural management solutions, nanotechnology has emerged as a promising alternative.^6^ This scientific and technological field, characterized by its multidisciplinary nature, involves the understanding, synthesis, control, manipulation, and application of materials at the nanoscale level. Nanomaterials are generally classified according to their morphologies, dimensions, and chemical compositions. Based on their dimensions, a nanomaterial must have at least one of its three spatial dimensions (length, width, or height) on the nanometer scale (10^–9^ m).^7^ Depending on these dimensional attributes, a nanostructure is categorized as 0D, 1D, 2D, or 3D, based on the dimension(s) of the material that are not within the nanoscale.^7^ Accordingly, 0D nanomaterials possess all dimensions at the nanoscale and are referred to as nanoparticles (NPs). 1D nanomaterials have one dimension, typically length, at the macroscale and include nanofibers, nanotubes, or nanowires. 2D nanomaterials have two dimensions, length and width, at the macroscale, forming structures known as nanosheets, and 3D nanomaterials are composed of assembled 0D, 1D, and/or 2D nanostructures, resulting in porous 3D architectures with dimensions at the macroscale^7^ (Figure 1).

**Figure 1:**
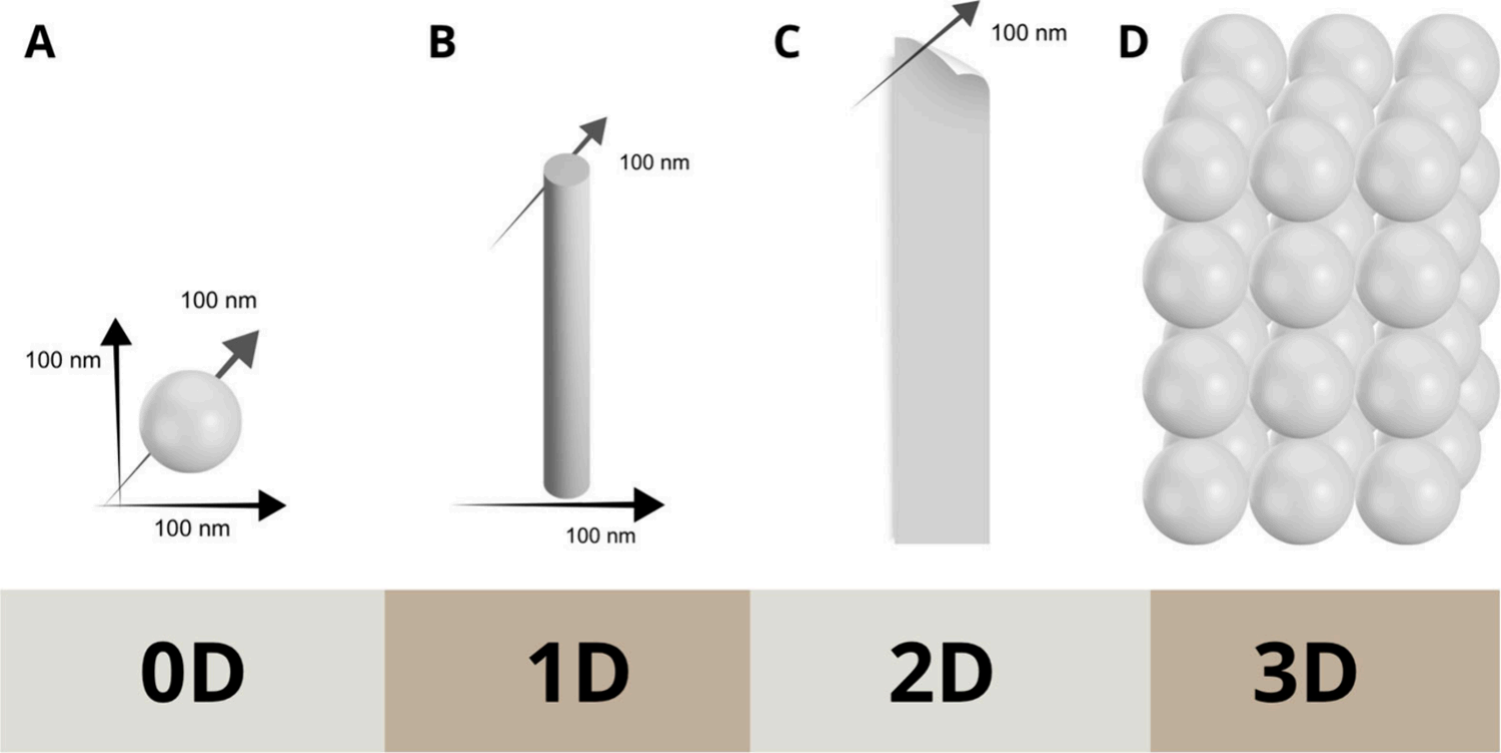
Classification of nanomaterials according to their dimensions (length, height, and width): (A) zero-dimensional (0D), (B) one-dimensional (1D), (C) two-dimensional (2D), and (D) three-dimensional (3D).

The morphological diversity of nanostructures available for the configuration of chemical elements, molecules, and complex biochemical structures enables a wide range of applications in nanotechnology. In agriculture, nanotechnology has the potential to enhance conventional plant production systems through the controlled release of agrochemicals such as fertilizers, pesticides, and herbicides, ensuring precise and environmentally safe application and increasing resistance to abiotic stresses.^8^ These improvements directly affect soil quality and lead to reduced water and energy consumption, resulting in more sustainable agricultural systems.^9–12^

NPs can be used as nanofertilizers, either incorporated into or applied as coatings on conventional fertilizers.^13,14^ Nanofibers and nanosheets may be employed to encapsulate conventional agrochemicals, enabling their slow release,^15–17^ whereas porous nanomaterials facilitate the controlled delivery of agrochemicals, moisture retention, and UV protection.^18,19^ The use of nanofertilizers effectively contributes to improved production quality, increased nutrient uptake efficiency, reduced costs, and minimized negative impacts on soil and water resources.^20^ For instance, nano-NPK formulations have demonstrated up to 25% higher nutrient use efficiency than conventional fertilizers in lettuce and maize trials.^21,22^ The selection of nanostructure types, application methods, and appropriate dosages for specific management practices is essential to achieve these outcomes.

A bibliometric analysis was conducted using the Web of Science Core Collection to better understand how these challenges have been addressed in the scientific literature (Supporting Information). The search was conducted in All Fields using the terms “nanofertilizer and nutrients” for the period from 2015 to 2025. A total of 181 peer-reviewed articles published over the mentioned period were analyzed using VOSviewer software. The co-occurrence network of author keywords revealed three major thematic clusters (Figure 2): (1) agronomic applications and nutrient management (green); (2) nanomaterials and application strategies (red); and (3) biological and environmental effects (blue). This thematic structure highlights the fragmented nature of the current research and underscores the need for integrated approaches that align scientific, policy, and industrial agendas.

**Figure 2:**
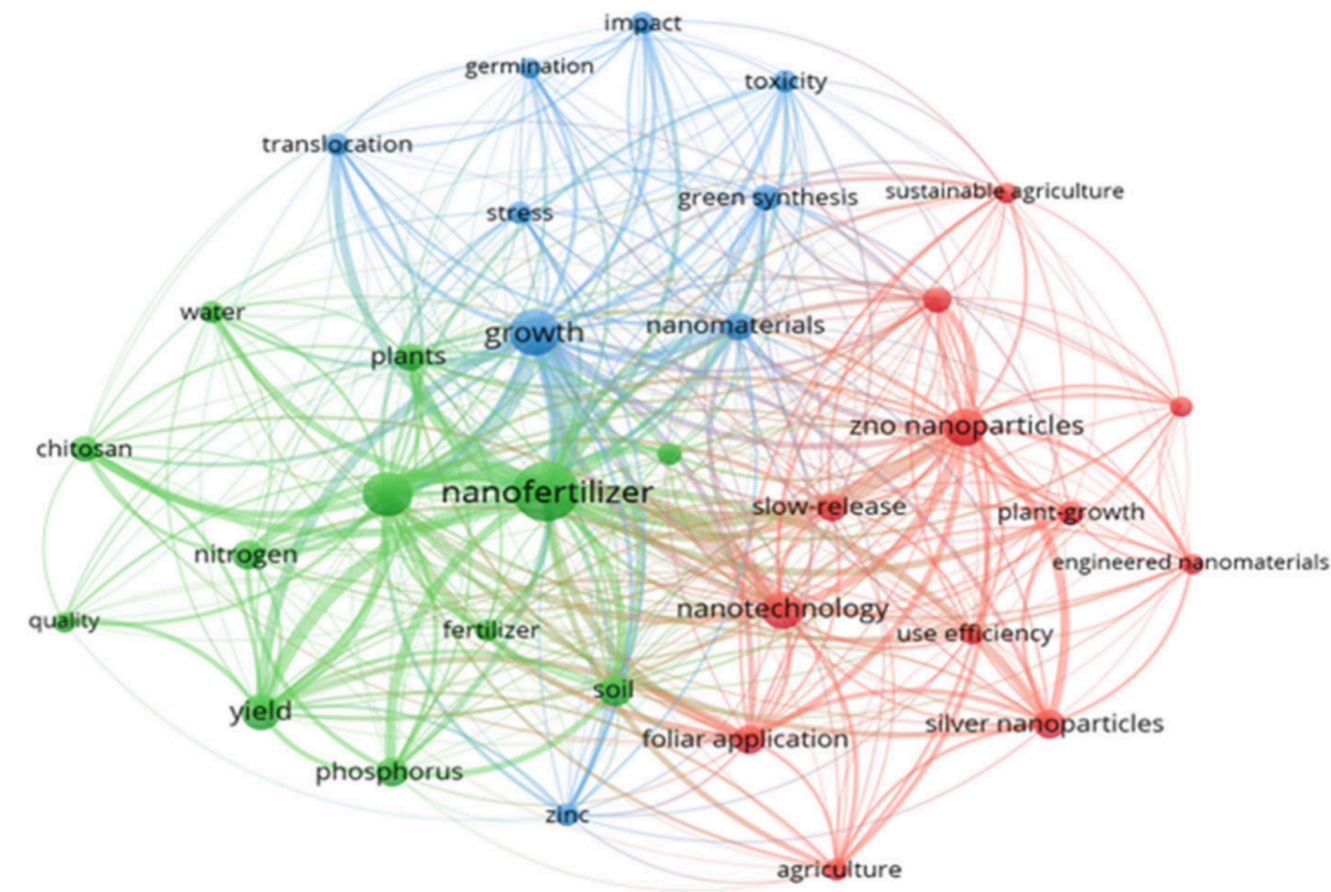
Co-occurrence network of author keywords from 421 articles on nanofertilizers in agriculture (2015–2025) based on data from the Web of Science Core Collection. The map was generated using VOSviewer with fractional counting and a minimum threshold of five keyword occurrences. Three main thematic clusters were identified: (1) agronomic applications and nutrient management (green); (2) nanomaterials and application strategies (red); and (3) biological and environmental effects (blue). The node size represents the frequency of each keyword, and the line thickness indicates the strength of the co-occurrence links between terms.

This review aims to provide a comprehensive overview of the current state of research on nanofertilizers by examining the main characteristics that distinguish them from conventional fertilizers, their properties, synthesis methods, mechanisms of absorption and transport, interactions with microorganisms and soil structure, as well as their agronomic benefits and challenges. In addition, we critically analyze the potential environmental risks, long-term uncertainties, and regulatory issues associated with the widespread use of nanomaterials in agriculture. A bibliometric analysis is presented to identify major publication trends and highlight the most recent advancements in the application of nanofertilizers to plant nutrition. By integrating these perspectives, this work seeks not only to consolidate and synthesize the existing body of knowledge but also to highlight the current research gaps that limit the application of nanofertilizers in sustainable agricultural practices. In particular, the review identifies critical priorities such as understanding long-term soil–plant–microbe interactions, evaluating the environmental fate of nanomaterials under field conditions, and developing standardized regulatory frameworks. Finally, this synthesis provides a roadmap for future investigations and helps align ongoing research with the broader goals of agricultural sustainability and global food security.

## 2 Nanomaterial Integration for Fertilizer Formulations

### 2.1 Concept of Nanofertilizers

Nanofertilizers are macro- and micronutrients that can be delivered to plants as particles or emulsions organized into structures or layers on the nanometer scale. These may include nutrients encapsulated or coated with nanomaterials as well as nutrients attached to nanoporous materials. Such configurations can function as protectants and carriers, imparting distinct dispersion, absorption, and translocation properties.^20,23,24^ Nanofertilizers can be classified according to plant nutritional demands, such as macro- and micronutrient-based nanofertilizers, organic nanofertilizers, hybrid nanofertilizers, carbon-based nanofertilizers, and nanobiofertilizers.^24,25^ Additionally, they may be categorized by the heterogeneity of their composition (monomeric or polymeric) and by their structural type (nanocapsules, nanocomposites, and nanodendrimers).^26^ This diversity allows nanofertilizers to be tailored to crop-specific nutritional needs and environmental conditions.^24,25^

One of the main advantages of nanofertilizers is their ability to minimize nutrient losses due to leaching, volatilization, and immobilization, which are the common drawbacks of conventional fertilizers. For instance, it is estimated that traditional formulations may lose up to 70–80% of the applied nutrients, depending on soil and climate conditions.^27^ By contrast, nanofertilizers provide a more targeted, stable, and efficient nutrient supply. Patil et al.^28^ reported yield increases above 20% in maize and wheat following foliar application of ZnO nanoparticles, along with reduced nutrient runoff under field conditions.

### 2.2 Properties of Nanomaterials and Nanofertilizers

Defined by having at least one dimension equal to or smaller than 100 nm, NPs exhibit distinct and enhanced physicochemical properties compared to their bulk counterparts, enabling more efficient applications.^29,30^ Properties such as antioxidant and catalytic activity, biocompatibility, permeability, structural attributes, and, most importantly, their nanoscale size make NPs suitable for applications across various fields, including biomedicine, biosensors, pharmaceuticals, and agriculture.^30,31^ Nanomaterials have a significantly higher surface-area-to-volume (SA/V) ratio compared to their bulk forms, allowing a greater number of surface atoms to be exposed and available for interaction, thereby greatly enhancing their reactivity with plant systems.^32^ Figure 3 illustrates the effect of particle size on the SA/V ratio. As the particle size decreases, the SA/V ratio increases exponentially, leading to more reactive surfaces for nutrient interactions and greater efficiency in uptake by plant roots and leaves.

**Figure 3:**
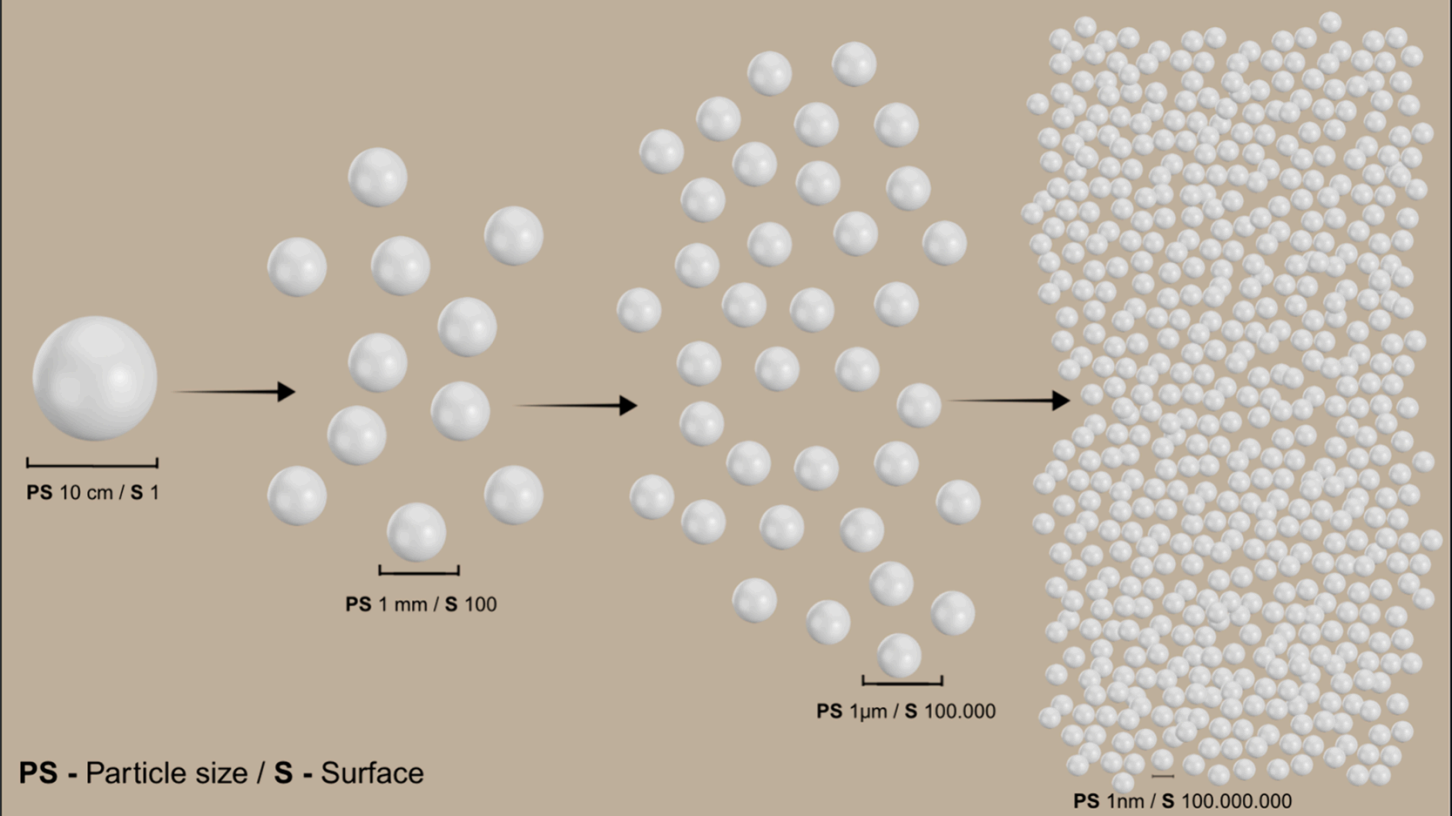
Comparison of the area-to-volume (SA/V) ratios of spherical particles of different diameters. As the particle size decreases to the nanoscale, the SA/V ratio increases exponentially, enhancing the chemical reactivity and interaction with plant tissues. This property is a key advantage of nanofertilizers over conventional formulations, as it improves nutrient availability and absorption efficiency.

The characteristics related to size, composition, and sensitivity confer nanofertilizers with various modes of interaction with plants. These interactions enhance plant growth, promote increases in fresh biomass, improve chlorophyll content, support metabolic activity, upregulate antioxidant potential, and enhance the expression of stress-related genes.^33^ In a study applying nanoencapsulated spermidine (an organic polyamine) to cabbage, Haghighi, Alviri, and Kappel^34^ observed a significant increase in the fresh weight of shoots and roots, a result attributed by the authors to the role of polyamines in promoting cell division and expansion. For instance, hybrid nanofertilizers composed of Zn and carbon dots have been shown to improve lettuce (*Lactuca sativa*) growth by enhancing light capture and photosystem II repair.^35^ The nanoscale also prevents the aggregation of otherwise unstable forms of nutrients. The basic units, molecules, or ionic crystalline structures of conventional fertilizers, such as urea, KCl, or P_2_O_5_, despite having molecular dimensions under 2 nm, can form aggregates during storage or in the soil, reducing their efficiency. Encapsulation in nanostructures (e.g., polyurethane matrices) preserves the nanometer scale and increases nutrient use efficiency.^36,37^ Figure 4 compares the molecular and nanoencapsulated forms of common fertilizers, illustrating how polymer coatings improve stability and allow controlled nutrient release. These properties explain why nanofertilizers often show 20–30% higher nutrient use efficiency than their conventional counterparts.^38^ Encapsulated fertilizers have been shown to release nutrients over 10–14 days, reducing environmental losses by up to 60% compared with conventional fertilizers.^20,39^

**Figure 4:**
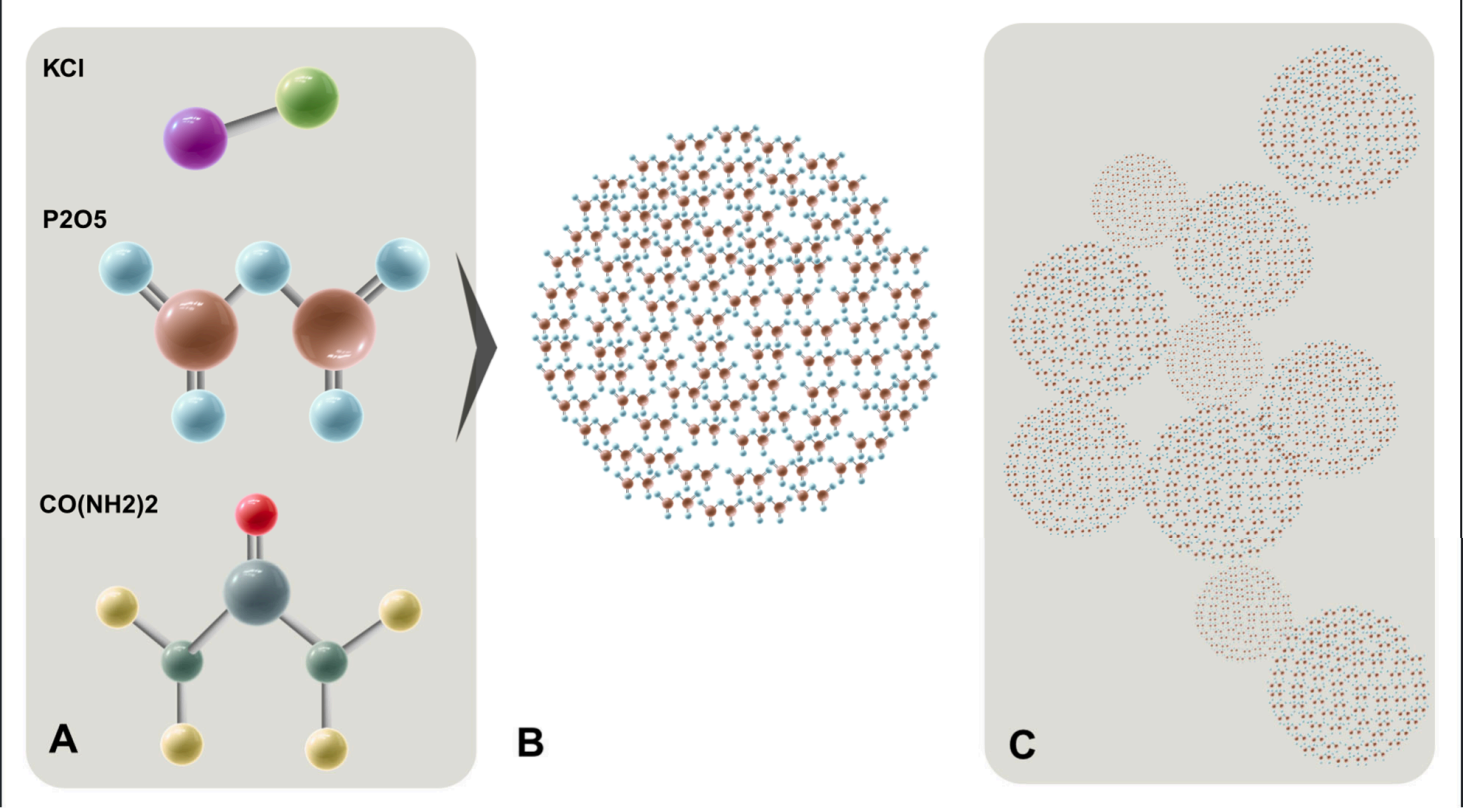
Comparison between conventional fertilizers and nanoencapsulated nutrient structures. (A) Molecular units of potassium chloride (KCl), phosphorus pentoxide (P_2_O_5_), and urea (CO­(NH_2_)_2_) with dimensions smaller than 2 nm. (B) Aggregated P_2_O_5_ forming unstable macrostructures that are prone to rapid dissolution and nutrient loss. (C) P_2_O_5_ encapsulated within a polymeric nanomatrix (e.g., polyurethane) with dimensions <100 nm, enabling controlled release, enhanced nutrient use efficiency (NUE), and reduced leaching.

### 2.3 Synthesis of Nanofertilizers

Nanofertilizers can be synthesized using top-down or bottom-up approaches. Top-down methods involve breaking down bulk materials into nanoparticles using physical or mechanical processes, often resulting in irregular particle sizes and high energy consumption.^40^ Bulk materials are broken down through different techniques such as sonication,^41–43^ ball milling,^43,44^ pulse discharge,^44,45^ and laser ablation.^46^ Bottom-up techniques, however, use chemical or biological processes to build nanoparticles from atomic or molecular precursors. Common methods include sol–gel synthesis,^47,48^ hydrothermal reactions,^48^ coprecipitation,^49^ and electrochemical deposition.^50^ While these methods provide better control over particle characteristics, they may involve toxic solvents or energy-intensive steps.^51^ Green synthesis methods have gained traction to address these issues.

#### 2.3.1 Green Synthesis

Green synthesis involves the use of biological organisms (e.g., plants, fungi, algae, and bacteria) as natural reducing and stabilizing agents to produce nanoparticles. These methods align with green chemistry principles by avoiding hazardous reagents and minimizing environmental impacts.^52,53^ Bioactive compounds, such as flavonoids, phenolics, alkaloids, and proteins, contribute to the reduction and stabilization of metal ions. For instance, extracts of *Azadirachta indica* have been used to synthesize Zn NPs that enhance millet growth and antioxidant responses.^54^ Among biological platforms, microalgae are particularly promising because of their high metabolic productivity and ease of cultivation. Figure 5 summarizes the green synthesis routes and typical stages of microalgae-mediated nanoparticle biosynthesis. Biosynthesis includes reduction, nucleation, and stabilization phases, which are typically driven by cellular metabolites and enzymes.^55,56^ Synthesis can occur extracellularly (on the cell wall surface) or intracellularly (within the cytoplasm) depending on the organism and conditions.^57,58^ Parameters such as pH, temperature, precursor concentration, and extract composition significantly affect the efficiency and properties of synthesized nanomaterials.^59,60^ However, challenges remain in achieving reproducibility and scalability in agricultural applications.

**Figure 5:**
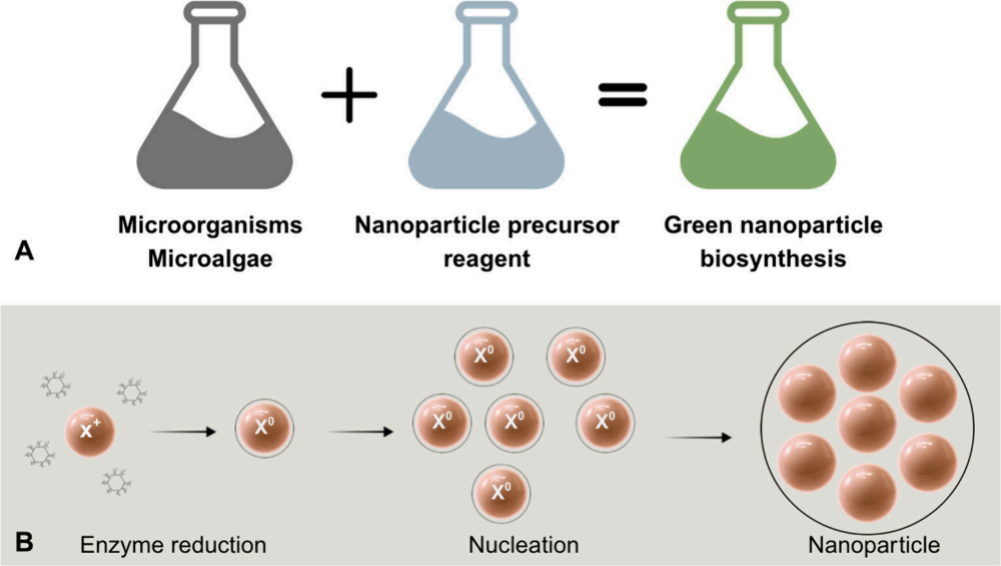
Overview of green synthesis routes for nanoparticle production. (A) Examples of bioactive compounds used in green synthesis, including phenolic compounds, alkaloids, flavonoids, and enzymes, extracted from plants, fungi, and bacteria. (B) Stages of microalgae-mediated biosynthesis illustrating algal growth, metabolite extraction, nanoparticle formation, and purification. These eco-friendly methods reduce energy consumption and avoid toxic byproducts, thus offering a sustainable alternative to conventional nanoparticle synthesis.

#### 2.3.2 Nanoencapsulation

Nanoencapsulation is a strategic technique in the development of nanofertilizers that enables the controlled and site-specific release of nutrients. By encapsulating active ingredients, such as nitrogen, phosphorus, or potassium, within nanostructured carriers, such as polymeric shells, liposomes, or mineral matrices, this approach enhances nutrient use efficiency (NUE) while reducing environmental losses.^20^ These nanoscale systems protect nutrients from degradation processes, such as volatilization, leaching, and microbial transformation, and allow for their gradual release in response to environmental stimuli, including soil moisture, temperature fluctuations, and root exudates.^61^ Field trials demonstrated the potential of this technology. For example, phosphorus pentoxide (P_2_O_5_) nanoencapsulated in polyurethane matrices resulted in a 70% reduction in phosphorus leaching compared with conventional superphosphate in rain-fed maize systems.^39^ Similarly, chitosan-based nanofertilizer composed of copper (Cu) and salicylic acid in maize seed treatment exhibited significantly antifungal activity and increased grain yield by 1.8-fold compared to the control.^62^ These results reinforce the advantages of nanoencapsulation for enhancing the stability and efficiency of nutrient formulations. Figure 4 illustrates the structural contrasts between conventional fertilizers and nanoencapsulated formulations, emphasizing the improved surface reactivity and functional stability of the nanomaterials. Such structural advantages, combined with the ability to fine-tune release kinetics and carrier composition, make nanoencapsulation a central innovation in the advancement of next-generation fertilizers.

## 3 Mechanisms of Action, Development, And Growth Promotion in Plants

### 3.1 Seed Priming and Treatment

The use of seeds with high physiological and sanitary qualities is essential to establish productive crops capable of expressing their full genetic potential. Parameters such as vigor, uniformity of emergence, and germination are closely associated with seed health and initial nutritional status. Seed treatment offers dual benefits: it protects against biotic and abiotic stressors while simultaneously providing essential nutrients that support early plant development.^63,64^ In the seed priming technique, seeds undergo controlled hydration and are immersed in nanofertilizer solutions. This process activates metabolic pathways linked to germination as nanofertilizers penetrate the seed coat through aquaporins and initiate internal dispersion.^65^ The resulting stimulation of growth-promoting hormones, mitigation of reactive oxygen species (ROS),^66^ and activation of antioxidant responses contribute to robust seedling establishment, even under adverse environmental conditions. Furthermore, priming minimizes nutrient leaching and enhances uptake efficiency, particularly in nutrient-deficient soils.^67^

Nanofertilizer-based seed treatments also contribute to early defense activation and nutrient mobilization.^68^ For example, a chitosan-based nanofertilizer containing copper and nanoencapsulated salicylic acid increased the activity of phenylalanine ammonia-lyase (PAL) and polyphenol oxidase (PPO), enzymes involved in systemic resistance, while protecting maize plants from postflowering stalk rot.^62^ The same study reported a 1.6-fold increase in the vigor index 10 days after germination, driven by elevated α-amylase and protease activities. Similar outcomes were observed when zinc-based nanoparticles synthesized using *Calotropis procera* extract were applied to pearl millet (*Pennisetum glaucum*). Nanoprimed seeds exhibited improved germination rates, vigor index, biomass accumulation, and photosynthetic efficiency.^54^ Similarly, titanium dioxide (TiO_2_) nanoparticles, when combined with humic acid and applied to lettuce seeds, enhanced antioxidant content and biomass, potentially by stimulating redox reactions that facilitate respiration during germination.^63^ Microorganism-mediated nanoparticle synthesis has also shown promise. Batool et al.^64^ demonstrated that *Enterococcus* sp. SR9-mediated silver nanoparticles (SR9AgNPs) enhanced the germination, biomass, and physiological parameters in wheat, cucumber, and tomato plants. Importantly, SR9AgNPs at 100 ppm were confirmed to be nontoxic, highlighting their potential for safe agricultural applications. These studies underscore the synergistic potential of nanomaterials and plant–microbe interactions in promoting early growth and defense responses.

### 3.2 Mechanism of Soil/Root Absorption and Transport

Soil application remains the most common method of fertilizer delivery, including nanofertilizers. Once in the soil matrix, nanofertilizers dissolve in water and are absorbed at the root tip or through root hair, often via aquaporin-mediated transport or endocytosis.^69^ After reaching the xylem, nanoparticles are translocated throughout the plant, rapidly improving their nutritional status.^67^

The root surface, which bears a net negative charge owing to mucilage secretion and the activity of root hairs, attracts positively charged nanoparticles, thereby facilitating uptake. This process is especially efficient in young tissues, where the epidermis and root hairs are not fully developed. Once in contact with the root surface, nanoparticles can enter via multiple mechanisms, including ionic exchange, protein–ligand interactions, endocytosis, or passive diffusion.^70,71^ Encapsulated nutrients benefit from a higher absorption efficiency and enhance enzymatic activity, including catalase and peroxidase stimulation.^72^ The efficiency of root uptake is influenced by the particle size. For example, Chen et al.^73^ demonstrated the uptake of 40 nm ZnO nanoparticles in rice seedlings, showing their presence in the root cortex and elongation zones. Similar findings in maize have confirmed translocation from roots to shoots.^74^

Nanoparticles may follow two principal transport pathways after they enter the root system. In the apoplastic route, they move between the cell walls and the plasma membrane, potentially reaching the endodermis unless blocked by the Casparian strip. Alternatively, in the symplastic pathway, transport occurs through the plasmodesmata, allowing passage from cell to cell and facilitating a broader systemic distribution (Figure 6B).

**Figure 6:**
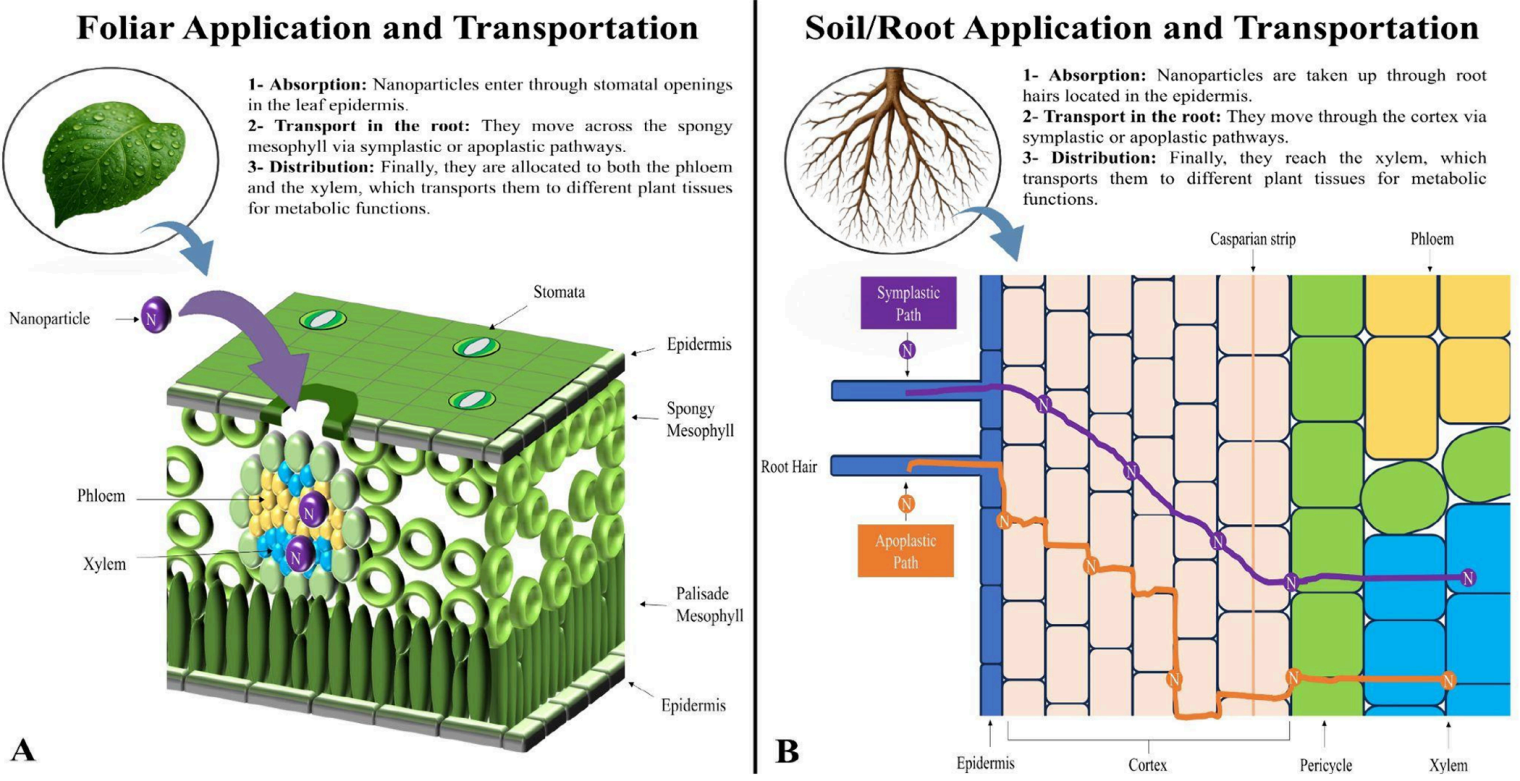
Pathways of nanoparticle (NP) uptake and transport pathways in plants. (A) Foliar application: NPs penetrate leaf tissues through the stomata, trichomes, cuticular cracks, and aqueous pores. After entering the mesophyll, NPs are translocated via symplastic or apoplastic routes and distributed through the phloem. (B) Soil and root application: NPs are absorbed by root epidermal cells via apoplastic and symplastic pathways, enter through cell walls, plasmodesmata, or endocytosis, and are transported to aerial parts via the xylem.

### 3.3 Mechanism of Foliar Absorption and Transport

Foliar fertilization offers a complementary strategy to soil fertilization and is particularly valuable under stress conditions or during periods of high nutrient demand. It allows for rapid correction of micronutrient deficiencies while avoiding soil-based nutrient imbalances.^2,75^ Nanoparticles applied via foliar spraying function as micronutrient sources, biostimulants, or protective agents. Their reduced size enables absorption through stomata, cuticular fissures, aqueous pores, trichomes, and ectodesmata. Once absorbed, nanoparticles are transported via diffusion and mass flow through the apoplast and symplast (Figure 6A), eventually reaching target tissues, such as roots, flowers, and fruits.^71,76^

The efficiency of foliar uptake is highly dependent on the nanoparticle size and charge. While stomatal openings can accommodate particles up to 50 nm, the foliar cuticle is far more selective, admitting particles only in the 0.6–4.8 nm range.^77^ Studies using *Lactuca sativa* and *Solanum lycopersicum* have confirmed the presence of Ag and Zn nanoparticles in the mesophyll tissues and vascular bundles following foliar application.^78,79^ Environmental variables, such as light, temperature, and humidity, also affect foliar uptake. High temperatures may cause epidermal contraction, whereas low humidity can induce stomatal closure, thereby limiting absorption. In contrast, high relative humidity enhances uptake by reducing osmotic potential, and high light intensity may increase stomatal openings, further facilitating absorption.^80,81^ Precipitation immediately after application can reduce foliar absorption by washing off nanoparticles from the leaf surface.^82^

### 3.4 Growth Promotion

The goal of nanofertilizer application is to enhance crop growth, yield, and nutritional quality. Macronutrients and micronutrients delivered in nanoscale formulations demonstrate greater bioavailability and responsiveness, which can lead to improved photosynthesis, enzymatic activity, stress tolerance, and biomass accumulation. Studies have demonstrated significant gains in plant growth with nanoformulations. For example, foliar application of nano-NPK at 3 mg L^–1^ increased vegetative growth in *Philodendron selloum*,^83^ while calcium nanoparticles at 200 mg L^–1^ improved the phytochemical content and yield in lettuce across two growing seasons.^84^ Magnesium nanofertilizers have also been proven to be more effective than conventional magnesium sulfate in enhancing the antioxidant capacity and nutrient density in common beans.^85^

Foliar applications of boron nanoparticles at 25–100 ppm increased nitrate reductase activity and stomatal conductance in beans while also improving biomass.^86^ These effects are summarized in the Supporting Information (Table S1), which summarizes nanofertilizer types, dosages, application methods, target crops, and observed benefits. By enabling precise nutrient delivery, enhancing plant metabolism, and reducing environmental loss, nanofertilizers represent a transformative tool for sustainable agriculture. Their adoption can substantially improve productivity while mitigating the ecological impacts of conventional fertilization.

### 3.5 Gradual Nutrient Release

One of the key advantages of nanofertilizers is their ability to provide a controlled and gradual release of nutrients, improve nutrient use efficiency (NUE), and reduce environmental losses caused by leaching, volatilization, or fixation.^72,87^ This mechanism is typically achieved through nanoencapsulation, surface adsorption, or the integration of smart release materials that respond to environmental stimuli such as pH, humidity, or root exudates. Biodegradable polymers such as chitosan, alginate, and polyurethane are widely used as encapsulating matrices, forming nanostructures that protect active ingredients and progressively degrade in the soil.^87,88^ This allows for sustained nutrient availability that better matches the temporal nutrient demands of the plants.^62^ For example, polyurethane-encapsulated P_2_O_5_ reduced phosphorus leaching by 70% in maize fields compared to conventional superphosphate under rain-fed conditions.^39^

Some nanocarriers have been designed to release nutrients in response to specific soil conditions. Changes in soil temperature, enzymes, and pH may induce structural changes or degradation of the nanostructure.^61^ Nutrient release can occur via multiple mechanisms, although it is commonly understood that it takes place primarily through diffusion across semipermeable membranes, where nutrients are gradually released in response to concentration or pressure gradients. The release rate of nanofertilizer contents is influenced by the nature of the polymer used, the concentration and viscosity of the solution, the number of coating layers, modifying agents, and the technique employed in constructing the nanostructure.^89^

This gradual release behavior contrasts sharply with that of traditional fertilizers, which typically discharge nutrients rapidly, often exceeding the uptake capacity of plants, leading to significant environmental contamination.^38^ Nanofertilizers represent a pivotal tool for precision agriculture, enabling crop-specific and environmentally conscious nutrient delivery.^85^ However, to fully realize these benefits, the formulation and environmental behavior of nanofertilizers must be optimized. The development of nanostructures requires attention to the material and method used, in addition to knowing the characteristics involved in the relationship between the material and the compound of interest.^90^ Therefore, future research should focus on tailoring release profiles to different crops, soil types, and climatic conditions to maximize agronomic gains while ensuring environmental safety.^91^

## 4 Applications of Nanofertilizers and Effects on Soil Structure and Health

### 4.1 Benefits of Nanoparticles on Soil Conditions

NPs offer significant benefits to the physical properties of soil, such as improved structure, porosity, and water retention, while also positively influencing fertility through the controlled release of nutrients and stimulation of microbial activity.^92–94^ Zinc oxide (ZnO) NPs enhance nutrient availability and soil fertility, thereby contributing to a more favorable environment for plant growth.^95,96^ Phung et al.^97^ demonstrated that the addition of ZnO NPs to composted sewage sludge had positive effects on soil fertility, promoting a significant increase in nitrogen (1.6 to 2.3 times) and potassium levels, essential elements for plant development. Furthermore, the presence of ZnO NPs increased the fractions of tyrosine-like substances and the biological index in the dissolved organic matter of the soil, indicating enhanced microbial activity and intensified nutrient cycling. These factors directly contribute to the improved quality and functionality of agricultural soils. Magnesium oxide (MgO) NPs, in turn, improve soil fertility by enhancing nutrient uptake, regulating soil pH, and contributing to plant tolerance against heavy metals and toxic metalloids.^96,98^

Nanoclays improve soil structure and aeration, increase nutrient availability, and stabilize contaminants, preventing their leaching into groundwater.^96,99^ Padidar et al.^100^ demonstrated that the application of nanoclays to sandy soils effectively controlled wind erosion. In wind tunnel experiments, the addition of 2000 ppm nanoclay increased the volumetric water content of the soil by 18.9% and reduced erosion losses by more than 99%, even at wind speeds of up to 67.3 km/h. There was also a 2.5% increase in the fraction of particles between 0.25 and 2 mm and a significant reduction (74%) in the fraction of particles smaller than 0.25 mm, indicating improved aggregation and structural stabilization of the soil.

Nanosilica (NPSiO_2_) has positive effects on soil structure, promoting greater water retention and enhanced nutrient delivery efficiency and pest control. It also contributes to pollutant immobilization, supporting long-term soil quality.^96,101^ According to Al-Saeedi,^102^ the application of NPSiO_2_ to soil resulted in significant improvements, with up to a 10-fold increase in the clay percentage using the highest dose (400 mg/kg). Porosity also increased significantly, reflecting the filling of macropores by NPs. Moreover, the saturation percentage increased by 36% compared with the control, indicating enhanced microporosity and water retention.

Carbon nanotubes (CNTs) offer benefits for soil strength and stability, supporting the controlled release of nutrients and herbicides.^96,103^ According to Alsharef et al.,^104^ incorporating small amounts (0.05–0.2%) of nanocarbons such as multiwalled CNTs and carbon nanofibers (CNFs) into residual soils significantly improved physical properties. These enhancements included increased pH (from 3.93 to 4.16), reduced hydraulic conductivity (from 2.16 × 10^–9^ to 7.44 × 10^–10^ m/s), increased maximum dry density, and reduced optimum moisture content and plasticity index, resulting in more stable soils less susceptible to deformation and water percolation. Thus, it is evident how different classes of nanoparticles can contribute individually or synergistically to the improvement of the physical conditions of different soils.

### 4.2 Nanoparticles in the Remediation of Contaminated Soils

The loss of soil health and functionality is a critical environmental issue that directly impacts agricultural productivity and ecosystem services.^105,106^ Various approaches have been employed to restore contaminated or degraded soils, among which the use of nanotechnology has gained increasing attention. NPs exhibit unique physicochemical properties, such as high surface area, enhanced reactivity, and the ability to interact with both organic and inorganic soil components.^93^

Iron oxide (Fe_3_O_4_, Fe_2_O_3_) NPs are widely used in the removal of heavy metals such as lead (Pb) and arsenic (As) through adsorption and reduction processes, directly contributing to improved soil quality.^96,107,108^ Singh et al.^109^ demonstrated that Fe_3_O_4_ NPs exhibit high adsorption capacities for metals such as cadmium (Cd), chromium (Cr), iron (Fe), nickel (Ni), copper (Cu), lead (Pb), and zinc (Zn). In contaminated soils and water, the removal efficiency ranged from 69.6% to 99.6% at pH 0.7 and from 63.5% to 98.3% at pH 4.5, with Pb being the most efficiently removed metal.

According to Zand et al.,^110^ the combination of titanium dioxide (TiO_2_) NPs with biochar promoted a greater growth of *Sorghum bicolor* and significantly enhanced the removal of antimony (Sb) from contaminated soils. Treatment with 250 mg/kg TiO_2_ NPs and 2.5% biochar resulted in the highest accumulation capacity (1624.1 μg/pot). Similarly, Bakshi and Kumar^111^ reported that the application of TiO_2_ NPs in association with the hyperaccumulator plant *Brassica juncea* was effective in removing Cd from soil. The NPs increased the plant’s tolerance to the metal, improving growth, biomass, and photosynthesis, with the highest removal efficiency (55.11%) observed at an NP concentration of 500 mg/kg. These findings highlight the potential of nanophytoremediation as a promising strategy for treating contaminated soils.

### 4.3 Effects on Soil Microbial Communities

The application of NPs to agricultural soils also has significant impacts on the rhizosphere microbiota and their functions, positively influencing plant growth and development.^88^ NPs act as mediators in plant–microbe interactions by modifying the rhizosphere environment in ways that favor the colonization and activity of beneficial microorganisms.^112,113^ These interactions can induce cooperation among microorganisms and the formation of homogeneous or heterogeneous biofilms, which enhance the protection and efficacy of bioinoculants under field conditions.^114^ Furthermore, coincubation of low concentrations of various types of nanomaterials can activate plant growth-promoting bacteria even under *in vitro* conditions, improving their functional traits and contributing to more effective soil performance.^114,115^

On the other hand, according to Civilini et al.,^116^ the application of cerium oxide (CeO_2_) NPs negatively influenced the bacterial composition of the rhizosphere of *Silene flos-cuculi*, particularly by reducing the abundance of anaerobic genera within the phylum Firmicutes. This shift affected key metabolic functions essential for soil fertility, such as biological nitrogen fixation, fermentation, and iron and nitrate respiration. In contrast, functions related to aerobic chemoheterotrophy were stimulated, indicating a potential redirection of microbial activity. These results suggest that CeO_2_ NP accumulation may impair essential soil biogeochemical cycles, thereby affecting nutrient availability and plant health.

According to Saleem et al.,^117^ the combined application of ZnO NPs and a biofertilizer containing zinc-solubilizing bacteria significantly improved wheat growth and zinc content in grains without causing substantial changes in the structure of the soil microbial community. In terms of microbial diversity, there was a marked increase in total aerobic bacteria (99%), fungi (34%), nitrogen-fixing bacteria (31%), phosphate-solubilizing bacteria (166%), and especially zinc-solubilizing bacteria (1400%). The population of Actinobacteria, however, remained unchanged. Illumina sequencing analysis revealed a relative increase in the abundance of *Bacillus*, *Massilia*, and *Rhizobium*, indicating a tendency to favor microorganisms with plant growth-promoting potential. Nevertheless, alpha diversity indices and ordination analyses suggest that despite these changes in relative abundances, the overall structure of the soil microbial community was not significantly altered.

Sun et al.^118^ revealed that selenium (Se) NPs, biosynthesized by selenium-associated bacteria from maize roots, may modulate soil microbiota like root exudates, promoting the recruitment of plant-beneficial microorganisms. *In vitro* and soil-based assays showed that Se NPs improved plant performance by stimulating, in a dose-dependent manner, colonization by plant growth-promoting bacteria such as *Bacillus*. Multiomics analysis revealed an inter-kingdom signaling cascade: maize roots detect histamine signals produced by *Bacillus* spp., which induce the exudation of *p*-coumarate by the plant. In response, the *rpoS* gene in selenium bacteria such as *Pseudomonas* sp. ZY71 is activated, promoting Se NP biosynthesis.

Nanofertilizers not only improve physical soil conditions, such as porosity and water retention, but also offer promising solutions for the remediation of contaminated soils by leveraging the unique properties of different NPs. These advances may lead to more sustainable and efficient agricultural practices. Additionally, the beneficial interactions between NPs and soil microbiota highlight their potential to promote plant health and growth without significantly disrupting existing microbial communities.

### 4.4 Regulatory and Safety Considerations

Despite the considerable benefits associated with the use of nanofertilizers in sustainable agriculture, their environmental fate, bioaccumulation potential, and long-term ecotoxicological effects remain poorly understood, particularly under field conditions. The increasing application of nanoparticles (NPs) in agroecosystems raises several safety concerns related to their persistence in the environment, toxicity to nontarget organisms, and occupational exposure risks, all of which are currently under-regulated in most countries.

One of the major challenges lies in the environmental persistence of metal-based NPs, such as ZnO NPs, TiO_2_ NPs, and AgNPs. These particles can remain in the soil matrix for extended periods, potentially altering microbial communities and physicochemical processes or leaching into groundwater, thereby increasing the risk of contamination and biomagnification in the food chain.^119^ Studies have shown that certain NPs, particularly at high doses, can adversely affect beneficial soil organisms, such as earthworms, mycorrhizal fungi, and nitrogen-fixing bacteria, disrupting essential ecosystem services, such as nutrient cycling and organic matter decomposition.^120–122^ Occupational health risks are also a growing concern, especially in scenarios involving the handling of dry or inadequately encapsulated formulations.^123^ Farmworkers may be exposed to these materials through inhalation or dermal contact, posing risks that are often overlooked owing to the absence of specific regulations or adequate personal protective equipment.^124^ From a regulatory perspective, most national and international frameworks have remained fragmented or outdated. In the European Union, nanomaterials are classified under the REACH regulation, guided by the precautionary principle; however, no dedicated protocols exist for nanofertilizers. The United States Environmental Protection Agency (EPA) has initiated voluntary reporting mechanisms, but comprehensive mandatory guidelines are lacking. In Brazil and other middle-income countries, agencies such as ANVISA and IBAMA have yet to implement consistent procedures for the classification, labeling, and risk assessment of nanoenabled agricultural inputs. The lack of harmonized international definitions and safety thresholds hampers product registration, environmental monitoring, and consumer awareness.

These gaps underscore the urgent need for standardized protocols for the risk assessment of nanofertilizers, considering their unique behavior in complex environmental matrices. Long-term field trials are essential for assessing ecological safety across different soil types, climates, and cropping systems. Life cycle assessment (LCA) tools should be applied to evaluate the sustainability and environmental trade-offs of these technologies, whereas certification and traceability schemes are necessary to enhance transparency and ensure regulatory compliance. Incorporating these regulatory and safety considerations into research, innovation, and policy design is critical for ensuring that nanofertilizers contribute to agricultural productivity without compromising environmental integrity, human health, or long-term sustainability.

## 5 Environmental Risks, Regulatory Frameworks, and Safety Considerations of Nanofertilizers

Nanofertilizers offer promising advantages for sustainable agriculture; however, their deployment raises significant concerns regarding environmental safety and regulatory oversight. Owing to their nanoscale dimensions and enhanced reactivity, these materials can interact with biological systems in unexpected ways, potentially leading to toxicological effects on nontarget organisms and ecological processes.

Numerous studies have shown that certain nanoparticles, particularly ZnO NPs, TiO_2_ NPs, and AgNPs, may induce oxidative stress, membrane damage, and genotoxicity in aquatic species such as *Daphnia magna*, algae, plants, and fish embryos.^125–128^ In terrestrial ecosystems, nanoparticles can persist in the soil, interact with microbial communities, and disrupt key functions such as nitrogen cycling and organic matter decomposition. For example, Civilini et al.^116^ observed that CeO_2_ nanoparticles altered the rhizospheric microbiota of *Silene flos-cuculi*, impairing critical metabolic pathways related to soil fertility. Similarly, Sun et al.^118^ demonstrated that selenium nanoparticles modulate microbial recruitment and biochemical signaling in maize rhizospheres, with complex implications for soil health. Despite these findings, the long-term environmental fate of nanofertilizers remains largely unknown. Data on their persistence, transformation, mobility, and bioaccumulation in soil–plant systems are scarce, and results often vary depending on environmental conditions and nanoparticle coatings. These knowledge gaps are compounded by the lack of standardized ecotoxicological testing protocols and inconsistent regulatory classification. From a governance perspective, regulatory frameworks addressing nanofertilizers are still incipient and vary widely across jurisdictions. The European Union applies the precautionary principle and regulates nanomaterials under REACH, yet it lacks specific guidelines tailored to nanofertilizers. In the United States, the Environmental Protection Agency (EPA) has proposed voluntary frameworks for nanomaterial registration, but binding requirements remain limited. Most low-income and middle-income countries do not have the institutional capacity to assess or regulate nanotechnology-based inputs in agriculture.^129^ As a result, many nanofertilizer products have been commercialized without clear labeling or traceability mechanisms, hindering risk management and consumer transparency.

Addressing the environmental risks and regulatory gaps associated with nanofertilizers requires coordinated actions on several fronts. First, harmonized risk assessment protocols that reflect the specific behavior of nanoparticles in agroecosystems must be developed. Second, regulatory definitions and classification criteria should be updated to explicitly encompass nanoproducts. Third, greater transparency regarding product composition and environmental labeling is essential for informing users and supporting monitoring efforts. Finally, an investment in interdisciplinary research and international collaboration is critical to ensuring that nanofertilizer technologies are both effective and environmentally responsible.

## 6 Challenges, Knowledge Gaps, and Research Priorities

Despite the growing interest in nanofertilizers as promising tools for sustainable agriculture, several critical challenges remain unaddressed. A major limitation is that most studies have been conducted under controlled laboratory or greenhouse conditions, which may not accurately represent the complex interactions that occur in field environments. Variables such as soil type, climate, irrigation regime, and crop species significantly influence nanoparticle behavior, affecting their efficacy, persistence, and potential risks in real agricultural settings.^130,131^ Another major gap lies in a limited understanding of the environmental fate, transformation, and bioaccumulation of nanoparticles. Although certain metal-based NPs may persist in soils or leach into water bodies, long-term field data on their degradation pathways and interactions with organic matter and soil colloids remain scarce. Particularly concerning is the possibility of nanoparticle uptake and translocation to edible plant tissues, which could lead to human exposure through the food chain. However, systematic evaluations of accumulation patterns and their potential health effects are rare.

Analytical limitations also hinder progress in this field. The detection, quantification, and speciation of nanoparticles in complex environmental matrices require sophisticated tools, such as single-particle ICP-MS, NanoSIMS, or synchrotron-based techniques, which are not widely accessible and lack standardized protocols for nanofertilizer-specific assessments. This limits the ability to compare results across studies and impairs the development of robust risk assessments. The interaction between nanofertilizers and rhizosphere microbiota represents another underexplored area. While some studies have reported beneficial effects on plant growth-promoting rhizobacteria,^132,133^ others have indicated potential disruption of microbial community structure and function.^133,134^ These divergent findings highlight the need for more detailed investigations using metagenomic and multiomics approaches to elucidate the mechanisms underlying such interactions. Finally, the absence of harmonized international regulations and definitions poses barriers to the safe deployment of nanofertilizers. Regulatory frameworks are fragmented with inconsistencies in product classification, labeling requirements, safety thresholds, and risk assessment protocols across countries. Without internationally agreed standards, both environmental monitoring and commercialization efforts remain limited. To address these gaps, future research should prioritize the following:

Long-term, multisite field trials under realistic agronomic conditions;
Mechanistic studies of nanoparticle–soil–plant–microbe interactions;
Development of accessible, standardized analytical methods for nanoparticle tracking;
Integration of LCA and exposure modeling into risk evaluation;
International cooperation for regulatory harmonization and data sharing.

Bridging these knowledge gaps is essential to unlocking the full potential of nanofertilizers while safeguarding the environment and human health.

## 7 Conclusions and Outlook

Nanofertilizers represent a transformative innovation in modern agriculture, offering the potential to enhance nutrient use efficiency (NUE), promote plant growth, and mitigate environmental degradation. Their unique physicochemical properties, such as high surface area, controlled release, and enhanced reactivity, enable precise nutrient delivery and reduce losses through leaching, volatilization, or immobilization. Furthermore, their integration into sustainable agricultural practices aligns with global goals of reducing chemical inputs and improving crop productivity under variable environmental conditions. Despite these promising advantages, the widespread adoption of nanofertilizers has been limited by several challenges. These include a limited understanding of their long-term environmental fate, the complexity of their interactions with soil and microbiota, and the risks posed to nontarget organisms and human health. Current regulatory frameworks are fragmented and insufficient to fully address the unique behaviors and potential hazards of nanoscale agricultural inputs. The lack of standardized definitions, testing protocols, and traceability mechanisms further hampers risk assessments and product approval across jurisdictions.

Future research should prioritize multidisciplinary approaches that combine agronomy, soil science, toxicology, and policy development. Long-term field studies are essential to evaluate efficacy, ecological safety, and potential trade-offs under real-world conditions. Simultaneously, LCA and ecotoxicological models should be incorporated into innovation pipelines to guide safe-by-design strategies for nanofertilizer development. Moreover, policy innovation is needed to harmonize regulatory practices and promote transparency across supply chains. The establishment of international guidelines, certification systems, and clear labeling requirements is vital to foster public trust and facilitate market entry. Equally important is the investment in education and extension programs to support informed decision making by farmers, agronomists, and stakeholders. In conclusion, nanofertilizers offer a promising pathway for more efficient and sustainable agriculture. However, their successful implementation depends on a holistic approach that balances technological innovation with environmental stewardship, regulatory clarity, and societal engagement.

## Supplementary Material



## Notes

Summary of nanofertilizer classifications, dosages, application methods, target crops, and achieved effects; bibliometric analysis including temporal evolution, geographical distribution, and analysis of similarities (PDF)

## Abbreviations

AgNP: silver nanoparticle
ANVISA: Brazilian Health Regulatory Agency
CeO_2_: cerium oxide
CNT: carbon nanotube
CO: carbon monoxide
EPA: Environmental Protection Agency (USA)
EU: European Union
Fe_2_O_3_: ferric oxide
Fe_3_O_4_: magnetite
IBAMA: Brazilian Institute of Environment and Renewable Natural Resources
LCA: life cycle assessment
NP: nanoparticle
NPK: nitrogen, phosphorus, potassium
NPSiO_2_: nanosilica
NUE: nutrient use efficiency
REACH: Registration, Evaluation, Authorisation and Restriction of Chemicals (EU)
TiO_2_: titanium dioxide
ZnO: zinc oxide
