# Supplementary material for: Nanofertilizers in Modern Agriculture: A Technological Revolution in Plant Nutrition and Resource Efficiency
Source: ACS Omega. 2025 Nov 21;10(48):58057–71. doi: 10.1021/acsomega.5c08087 (PMC12771263; doi:10.1021/acsomega.5c08087)
Supplement: Supplementary file 1 [file ao5c08087_si_001.pdf]

Nanofertilizers in modern agriculture: A technological revolution in plant nutrition and resource efficiency

*Moacir C. do Couto Junior [1]; Leandro I. da Silva [2]; Mariana de S. Ribeiro [1]; Caroline Dambroz [1]; Tatiana C. e Bufalo[3]; Marcelo Pedrosa Gomes[4] and Joyce Dória[1, *]*

[1]Department of Agriculture, Federal University of Lavras (UFLA), Lavras 37203-202, Brazil; moacir.junior@estudante.ufla.br (M.C.C.J.); marianadsr20@gmail.com (M.S.R.); carolinedambroz@gmail.com (C.D.)

[2]Department of Biology, Federal University of Lavras (UFLA), Lavras 37203-202, Brazil; leandro.silva14@estudante.ufla.br (L.I.S.)

[3]Department of Physics, Federal University of Lavras (UFLA), Lavras 37203-202, Brazil; tatiana.cardoso@ufla.br (T.C.e B.)

[4]Department of Botany, Biological Sciences Sector, Federal University of Paraná, Avenida Coronel Francisco H. dos Santos, 100, Polytechnic Center, Jardim das Américas, P.O. Box 19031, 81531-980, Curitiba, Paraná, Brazil.

* Correspondence: joyce.doria@ufla.br

Supporting Information 1

Table S1. Summary of nanofertilizer types, concentrations, application strategies, and their agronomic and physiological effects on different crops.

Nanofertilizer (classification)	Dosages	Application Methods	Crop	Plant-Growing Condition	Achieved Effects	References
Organic Nanofertilizer (Spermidine)	1mM	Foliar spray	Cabbage *(Brassica oleracea* spp.)	Stress free (Glasshouse condition)	Enhance nutritional content and antioxidant properties	1
Hybrid Nanofertilizer (L-aspartate nano-calcium)	150 mg/L	Nutrient solution	Tobacco *(Nicotiana tabacum L.)*	Stress free (Artificial climate chamber)	Beneficial for growth and utilization of potassium.	2
Hybrid Nanofertilizer (Zinc-Carbon Dot)	10 mg/L	Foliar spray	Lettuce *(Lactuca sativa L.)*	Stress free (Greenhouse experiment)	Enhance the photosynthetic activity; Improve multiple-nutrient uptake; Regulate the rhizosphere soil environment; Increase lettuce yield and quality.	3
Micronutrient Nanofertilizer (Mg(OH)2 NPs)	1 mmol/L	Hydroponic Seedlings	Tomato *(Solanum lycopersicum L.)*	Stress free (Hydroponic growth chamber)	Effectively mitigate the antagonistic effects of K on Mg.	4
Micronutrient Nanofertilizer (ZnO NPs)	50 ppm, 100 ppm, 150 ppm, 20 ppm	Seed priming	Pearl millet *(Pennisetum glaucum)*	Stress free (Laboratory and greenhouse conditions)	Improvement in % germination potential, vigour index and agronomic traits such as plant height, dry weight, fresh weight, leaf area, photosynthetic pigments, and antioxidant activity.	5

Nanofertilizer (classification)	Dosages	Application Methods	Crop	Plant-Growing Condition	Achieved Effects	References
Micronutrient Nanofertilizer (Se and Cu NPs)	100 mg/L	Foliar spray	Strawberry *(Fragaria x ananassa)*	Stress (Under drought stress)	Increased the agronomic traits of strawberry plants, improved fruit quality and enhanced the content of photosynthetic pigments (chlorophyll a, chlorophyll b, and total chlorophyll), photosynthetic characteristic parameters, chlorophyll fluorescence parameters, and water-use efficiency.	6
Micronutrient Nanofertilizer (Fe2O3 NPs)	20 µg/mL	-	Eggplant *(Solanum melongena)*.	Stress (*Fusarium oxysporum established*)	Beneficial effects on the activity of antioxidant enzymes, osmolytes, comprising soluble sugar, proline, and soluble protein and photosynthetic pigments.	7
Chitosan NPs	-	Soil application	Camelina *(Camelina sativa L.)*	Stress (Under rainfed and irrigated conditions - supplementary irrigation)	Enhanced resistance to drought stress, plant growth. Increased relative water content, SPAD index and production of enzymatic and non-enzymatic antioxidants. Improve grain yield, oil contet and quality.	8
Macronutrient Nanofertilizer (Ca NPs)	200 mg/L	Foliar spray	Lettuce *(Lactuca sativa L.)*	Stress free (Field conditions - Agricultural management for lettuce plants were applied)	Improved agrophysiological characteristics, quality, and the content of minerals, phytochemicals, and antioxidants.	9

Nanofertilizer (classification)	Dosages	Application Methods	Crop	Plant-Growing Condition	Achieved Effects	References
Biological Nanocompósite (ZnMgO2 NPs)	25 and 75 mg/L	Seed priming	Rice *(Oryza sativa)*	Stress free (Laboratory and hydroponic conditions)	Promote shoot and root growth. Enhanced α-amylase activities, total phenol and flavonoid contents, and total antioxidant activities.	10
Biological Nanofertilizer (CuO NPs)	50 mg/kg and 40 ppm	Soil and foliar application	Cowpea *(Vigna unguiculata L. Walp.)*	Stress free (Experiment conducted in pots during the growing season)	Gains in leaf area, number of leaves, fresh and dry weights of shoots and roots, and the yield extract. Substantial effect on the non-enzymatic antioxidant concentrations of plants. Affected the number of photosynthetic stains, including carotenoids, chlorophyll *b*, and chlorophyll *a*.	11
Micronutrient Nanofertilizer (B NPs)	50 and 100 mg/L	Foliar spray	Potato *(Solanum tuberosum L.)*	Stress free (Field experiment - The standard agronomics practices for potato were applied)	Significantly improved the photosynthetic pigments except the anthocyanin content. Improved the contet of N and B and the total tuber yield, and significantly reduced tuber cracking percentage.	12
Hybrid Nanofertilizer (Ca-doped and ZnO NPs)	1 mg/100ml	Seed treatment and foliar spray	Maize *(Zea Mays L.)* and Wheat *(Triticum aestivum L.)*	Stress free (Field experiment)	Impact on seed germination, root and shoot length, plant height, root and stem width, number of leaves, and leaf size.	13
Macronutrient Nanofertilizer (NPK and Chitosan)	3 mg/L	Foliar application	*Philodendron sellum*	Stress free (Pot study under plastic house condition)	Increased vegetative growth parameters, root growth and chemical constituents.	14

Nanofertilizer (classification)	Dosages	Application Methods	Crop	Plant-Growing Condition	Achieved Effects	References
Micronutrient Nanofertilizer (Zinc-exchanged montmorillonite clay)	-	Soil application	Rice *(Oryza sativa)*	Stress free (Pot study under greenhouse condition)	Significantly high plant height, leaf area index, dry matter production, number of tillers per hill, panicles length, increased grain and straw yield. Increased total phenol, total protein and total chlorophyll content and phytochemicals as IAA, SOD, CA.	15
Hybrid Nanofertilizer (Cu, Zn and urea hydroxyapatite)	50 mg/plant	Soil application	Lemon *(Citrus limon)*	Stress free	Improved growth rate and brighter colored leaves.	16
Macronutrient Nanofertilizer (Nano-diammonium phosphate)	-	Foliar application	Rice *(Oryza sativa)*	Stress free (Field experiment condition)	Optimized physiological and biochemical traits, enhanced crop performance indicated by higher yield. Improved NUE.	17
Hybrid Nanofertilizer (NPK, Fe, Zn nanocomposite – chitosan nanographene)	-	Soil application	Tomato *(Solanum lycopersicum L.)*	Stress free (Pot study under greenhouse condition)	Increased the plant growth parameters including the plant height and the plant fresh and dry weight, as well as the plant chlorophyll content. Increased total shoot nitrogen, shoot phosphorus, shoot potassium, shoot iron, and shoot zinc content	18
Organic Nanofertilizer (CaCO3 NPs)	10 and 30 ppm	Foliar spray	Tomato *(Solanum lycopersicum L.)*	Stress free	Enhanced the total carotenoid level and essential nutritional minerals in fruit yield of tomatoes.	19

REFERENCES

1 - Haghighi, M., Alviri, A., & Kappel, N. Screening of spermidine-mediated improvement of plant growth, photosynthetic performance, metabolites, and nutrition acquisition in six cabbage varieties. *South African Journal of Botany*, **2025**, *178*, 50-60. https://doi.org/10.1016/j.sajb.2025.01.021

2 - Chen, X., Begum, N., Kong, D., Gong, Y., Ahmad, M., Wang, P., ... & Zhang, L. The appropriate concentration of L-aspartate nano-calcium promotes growth and potassium utilization in cigar varieties of tobacco (*Nicotiana tabacum L.*). *Rhizosphere*, **2025**, *33*, 101009. https://doi.org/10.1016/j.rhisph.2024.101009

3 - Ren, Y., Zhang, D., Cheng, B., Chen, B., Yue, L., Cao, X., ... & Wang, Z. Foliar Spraying Zinc–Carbon Dot Nanofertilizer Promotes Yield and Quality of Lettuce (Lactuca sativa L.) through Leaf–Root Regulation. *ACS Agricultural Science & Technology*, **2025**. https://doi.org/10.1021/acsagscitech.4c00651

4 - Chen, W., Hu, Z., & Liu, D. Compared to MgSO4, the use of magnesium nanofertilizer alleviates potassium-magnesium antagonism in tomato roots. *Agriculture*, **2025**, *15*(4), 368. https://doi.org/10.3390/agriculture15040368

5 - Kumar, R., Dadhich, A., Dhiman, M., Sharma, L., & Sharma, M. M. Stimulatory effect of ZnO NPs as a nanofertilizer in seed priming of pearl millet (Pennisetum glaucum) and their bioactivity studies. *South African Journal of Botany* **2024**, *165*, 30-38. https://doi.org/10.1016/j.sajb.2023.12.001

6 - Liu, A., Xiao, W., Lai, W., Wang, J., Li, X., Yu, H., & Zha, Y. Potential application of selenium and copper nanoparticles in improving growth, quality, and physiological

characteristics of strawberry under drought stress. *Agriculture*, **2024**, *14*(7), 1172. https://doi.org/10.3390/agriculture14071172

7 - Elbasuney, S., El-Sayyad, G. S., Abdelaziz, A. M., Rizk, S. H., Tolba, M. M., & Attia, M. S. Stable colloidal iron oxide nanoparticles: A new green nanofertilizer and therapeutic nutrient for eggplant immune response against fusarium wilt disease. *Journal of Cluster Science*, **2024**, *35*(4), 983-997. https://doi.org/10.1007/s10876-023-02527-3

8 - Haghaninia, M., Javanmard, A., Kahrizi, D., Bahadori, M. B., & Machiani, M. A. Optimizing oil quantity and quality of camelina (Camelina sativa L.) with integrative application of chemical, nano and bio-fertilizers under supplementary irrigation and rainfed condition. *Plant Stress*, **2024**, *11*, 100374. https://doi.org/10.1016/j.stress.2024.100374

9 - Salama, D. M., Osman, S. A., Mahmoud, S. H., El-Tanahy, A. M., & Abd El-Aziz, M. E. Improving the Productivity and Physiological Characteristics of Lettuce Plants Using Spraying Calcium as a Nanofertilizer. *Horticulturae* **2024**, *10*(11), 1157. https://doi.org/10.3390/horticulturae10111157

10 - Mehmood, S., Kumar, N., Mansoori, A., Mohan, M., Kumar, A., & Ghorai, T. K. Effect of ZnMgO 2 nanoparticles used as a nanofertilizer: promoting the growth activities of rice seedlings. *Environmental Science: Nano*, **2024**, *11*(4), 1571-1581. https://doi.org/10.1039/D3EN00770G

11 - Mustafa, M., Azam, M., Bhatti, H. N., Khan, A., Zafar, L., & Abbasi, A. M. R. Green fabrication of copper nano-fertilizer for enhanced crop yield in cowpea cultivar:

A sustainable approach. *Biocatalysis and Agricultural Biotechnology* **2024**, *56*, 102994. https://doi.org/10.1016/j.bcab.2023.102994

12 - Dhiman, D., Kalia, A., Sharma, S. P., Taggar, M. S., & Dheri, G. S. Nano-boron foliar application reduced the proportion of cracked tuber yield in potato. *Biocatalysis and Agricultural Biotechnology*, **2024**, *58*, 103182. https://doi.org/10.1016/j.bcab.2024.103182

13 - Patil, B. M., Patil, V. L., Bhosale, S. R., Bhosale, R. R., Ingavale, D. R., Patil, S. S., ... & Vanalakar, S. A. Field application of Ca-doped ZnO nanoparticles to maize and wheat plants. *Plant Physiology and Biochemistry*, **2024**, *210*, 108552. https://doi.org/10.1016/j.plaphy.2024.108552

14 - Nofal, E., Menesy, F., M Abd El-Hady, W., G Shehab, E., El-Ramady, H., & Prokisch, J. Effect of Nano-NPK and Nano-Chitosan Fertilizers on the Growth and Chemical Constituents of Philodendron sellum Plants. *Egyptian Journal of Soil Science* **2024**, *64*(3), 1193-1205. 10.21608/EJSS.2024.289119.1769

15 - Cyriac, J., Sreejit, C. M., Yuvaraj, M., Joseph, S., Priya, R. S., Saju, F., & Thomas, B. Zinc-exchanged montmorillonite clay: A promising slow-release nanofertilizer for rice (Oryza sativa L.). *Plant Physiology and Biochemistry*, **2024**, *212*, 108790. https://doi.org/10.1016/j.plaphy.2024.108790

16 - Waheed, Z., Anwar, A., Sadiqa, A., Ahmad, A., Intisar, A., Javaid, A., ... & Kazi, M. Advancing sustainable agriculture: Metal-doped urea–hydroxyapatite hybrid nanofertilizer for agro-industry. *Nanotechnology Reviews*, **2024**, *13*(1), 20240107. https://doi.org/10.1515/ntrev-2024-0107

17 - Sweety, R. R. B., Boominathan, P., Senthil, A., Jegadeeswari, D., & Kumar, G. P. Nano-diammonium phosphate enhances grain yield by modulating gas exchange traits and nutrient use efficiency in rice. *Plant Science Today*, **2024**, *11*, 5508. https://doi.org/10.14719/pst.5508

18 - Raiesi Ardali, T., Ma'mani, L., Chorom, M., Motamedi, E., & Fathi Gharebaba, M. A biocompatible NPK+ Fe+ Zn slow-release fertilizer: synthesis and its evaluation in tomato plant growth improvement. *Scientific Reports*, **2024**, *14*(1), 4640. https://doi.org/10.1038/s41598-024-55152-z

19 - Nallasamy, P., & Natarajan, S. Organic fertilizer integrated with marine waste derived CaCO3 nanocarrier system: A focus on enhanced yield and quality in tomato cultivation. *Scientific Reports* **2024**, *14*(1), 25299. https://doi.org/10.1038/s41598-024-70478-4

Supporting Information 2

Materials and Methods

1. Data Analysis: Research Method and Selection

The data compiled in this review were collected from the Web of Science platform in May 2025. The search was conducted in All Fields using the terms "nanofertilizer and nutrients" for the period from 2015 to 2025. A total of 181 documents were retrieved and exported as tab-delimited text files, with each record containing information on the title, authors, affiliations, keywords, and all citation data.

Additionally, from the Web of Science data, information was extracted and tabulated regarding the number of publications over the evaluated years and the countries with the highest number of scientific contributions in this field, measured by the publication count during the same period.

2. Bibliometric Review Methodology

A bibliometric analysis was conducted using the Web of Science Core Collection to identify key trends and research gaps related to nanofertilisers. The search included peer-reviewed articles published in English between January 2015 and May 2025. The strategy uses six thematic blocks of keywords connected using Boolean logic:

1. Nanofertilizers and nanotechnology in agriculture: "nanofertilizer", "nanoparticles", "nanotechnology", "nano-enabled inputs"

2. Plant nutrition and nutrient use efficiency: "nitrogen use efficiency", "phosphorus", "micronutrients", "slow-release fertilizers"

3. Soil and environmental interactions: “soil health”, “soil microbiota”, “bioavailability”, “soil amendments”

4. Ecotoxicology and environmental fate: “ecotoxicity”, “bioaccumulation”, “environmental fate”, “toxicity”

5. Regulatory and safety aspects: “regulatory framework”, “risk assessment”, “governance”, “occupational exposure”

6. Sustainability and green synthesis: “green synthesis”, “sustainability”, “biocompatibility”, “life cycle assessment”

The keywords within each block were connected using the Boolean operator OR, whereas the blocks were combined using AND. After removing duplicates and screening abstracts, 421 articles were selected. Bibliometric mapping was performed using VOSviewer using the fractional counting method. A threshold of 10 keyword occurrences was applied to enhance the focus on the major research trends. After manual standardization and cleaning, 49 keywords were retained to construct a co-occurrence network (Table S2).

Table S2. Description of the selected keywords in the visualization of the similarities analysis conducted on publications about nanofertilizers and agriculture between 2015 and 2025.

Keyword	Occurrences	Total Link Strength
Nanofertilizer	92	326
Growth	53	225
NPs	48	204
Nanotechnology	35	133
Zno NPs	34	164
Yield	30	119
Soil	22	86
Foliar application	21	109
Nitrogen	21	72
Silver NPs	21	86
Phosphorus	20	73
Nanomaterials	18	96
Plants	18	92
Slow-release	18	77
Chitosan	15	60
Green synthesis	15	75
Oxide NPs	15	75
Plant-growth	13	58
Agriculture	12	59
Fertilizer	12	42
Stress	12	57
Toxicity	12	42
Translocation	12	53
Water	12	44
Zinc	12	53
Impact	11	51
Phytotoxicity	11	56
Use efficiency	10	49
Engineered nanomaterials	9	42
Germination	9	47
Hydroxyapatite NPs	9	37
Quality	9	28
Sustainable agriculture	9	53

Note: A total of 421 articles were included in this bibliometric review. Keywords were extracted, standardized, and filtered to remove duplicates and synonyms. A co-

occurrence map was generated using VOSviewer (v.1.6.19) by applying fractional counting to enhance the interpretability of the keyword network

Results and Discussion

1. Temporal Evolution of Scientific Publications

According to the data presented in Figure S1, there was a clear upward linear trend in the number of research articles related to nanotechnology and agriculture published over the years. Notably, the combined publications from 2015 to 2018 were relatively few (n = 13), with the primary focus of these early publications on nanotechnology as an innovation in fertilization. Subsequent studies have shifted their emphasis toward topics such as sustainability, efficiency, and practical applications in crop production.

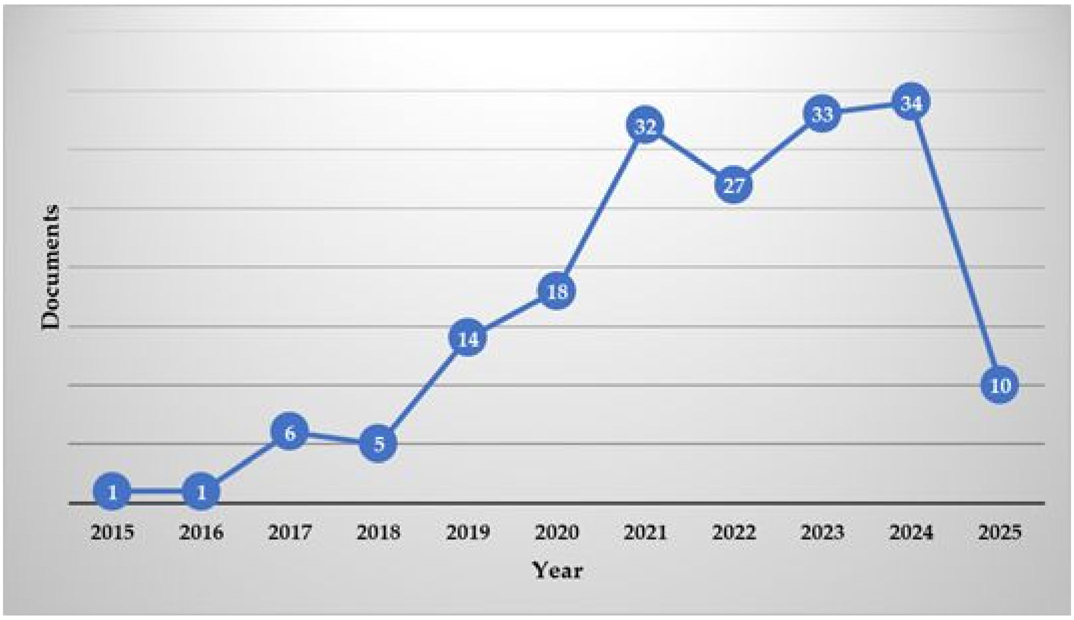

Figure S1. Number of publications on nanofertilizers and agriculture between 2015 and 2025.

The year 2019 can be considered a milestone in the shift of research topics, as its main thematic foundations have been established and maintained until the present day. This year alone recorded more publications than all previous years combined (n = 14),

with key themes indicating diversification in synthesis types, processing methods of nanofertilisers, applications in agriculture and sustainability, and impacts on soil and crops. Subsequently, 2021, 2023, and 2024 exhibited the highest number of publications in the bibliographic survey, collectively accounting for 99 documents, representing 54.7% of the total works retrieved. These data reflect the positioning of nanotechnology as a priority and scientifically relevant field, leading to a global increase in research incentives and underscoring the growing interest in the application of nanotechnology to agriculture.

2. Geographical Distribution of Scientific Publications

The geographical distribution of publications is presented in Table S3. India has a wide margin of 62 publications, representing more than twice the number of second-ranked countries in the United States (29). The prominence of India can be attributed to factors such as its high dependence on agriculture in its economy, government incentives for research in nanotechnology applied to agriculture, and the pursuit of low-cost solutions for efficient fertilization. The country has the second-largest area of agricultural land worldwide, with 157.35 million hectares under cultivation, spanning 20 distinct agro-climatic zones 1,2. The presence of the USA and China, global scientific powerhouses, confirms the role of nanofertilisation as a topic of strategic international interest.

Table S3. Countries and number of publications on nanofertilizers and agriculture between 2015 and 2025.

Country	Number of Studies
India	62
USA	29
China	21
Egypt	16

Pakistan	12
Iran	10
Mexico	10
Hungary	8
Italy	7
Philippines	7
Saudi Arabia	7
South Korea	7
Spain	7
Brazil	5
Nigeria	5

Countries in the Middle East and North Africa, such as Egypt, Iran, and Saudi Arabia, also demonstrate growing involvement, possibly driven by the need for solutions to address degraded soils and arid climates 3.

Europe, particularly Hungary, Italy, and Spain, has made significant contributions, whereas Brazil accounted for only five studies during the analyzed period, reflecting an underexplored potential despite the importance of agriculture to its economy. According to some references 4, BRICS countries (Brazil, Russia, India, China, and South Africa) have state-led initiatives in nanotechnology, with notable scientific advances but limitations in innovation. Institutional differences, socioeconomic challenges, and geopolitical factors affect policy formulation and outcomes, resulting in distinct trajectories even among countries with similar structures. The presence of Nigeria, although modest, indicates the beginning of research efforts in African countries with a high demand for agricultural innovation 5.

REFERENCES

1 - Eliazer Nelson, A.R.L.; Ravichandran, K.; Antony, U. The impact of the Green Revolution on indigenous crops of India. *J. Ethn. Food* **2019**, *6*, 8. https://doi.org/10.1186/s42779-019-0011-9

2 - Paul, B.; Patnaik, U.; Sasidharan, S.; Murari, K.K.; Bahinipati, C.S. Fertilizer Use, Value, and Knowledge Capital: A Case of Indian Farming. *Sustainability* **2022**, *14*, 12491. https://doi.org/10.3390/su141912491

3 - Qader, S.H.; Dash, J.; Alegana, V.A.; Khwarahm, N.R.; Tatem, A.J.; Atkinson, P.M. The Role of Earth Observation in Achieving Sustainable Agricultural Production in Arid and Semi-Arid Regions of the World. *Remote Sens.* **2021**, *13*, 3382. https://doi.org/10.3390/rs13173382

4 - Ramos Torres, C.; Invernizzi, N. Evolution of nanotechnology policy initiatives in the BRICS countries: a comparative overview. *TransAmerica Review* **2024**, *2*, e24007. https://doi.org/10.62910/transame24007

5 - Adeyemi, S.O.; Sennuga, S.O.; Bamidele, J.; Alabuja, F.O.; Omole, A.O. A critical review of rural agricultural development innovative programmes in Nigeria. *Plant Biol. Soil Health J* **2023**, *1*, 11–17.
